# Microarrays—Current and Future Applications in Biomedical Research

**DOI:** 10.3390/microarrays1010042

**Published:** 2011-11-22

**Authors:** Ulrich Certa

**Affiliations:** 1 Editor-in-Chief of Microarrays, Molecular Toxicology, Non-Clinical Safety (pRED), F. Hoffmann-La Roche AG, Postfach, 4070-Basel, Switzerland; Email: ulrich.certa@roche.com

**Keywords:** n/a

## Abstract

*Microarrays* covers research where microarrays are applied to address complex biological questions. This new open access journal publishes articles where novel applications or state-of-the art technology developments in the field are reported. In addition, novel methods or data analysis algorithms are under the scope of *Microarrays*. This journal will serve as a platform for fast and efficient sharing of data within this large user community. As one of the first microarray users in Europe back in 1996, I am proud to serve as Editor-in-Chief and I believe we have assembled a highly proficient Editorial Board, responsible for a fair and fast peer-review of articles.

The first generations of commercial microarrays introduced in the mid-nineties suffered mainly from sensitivity and reproducibility due to poor production technologies. In the early days of the technology, the first microarrays had probe sets complementary to about 120 genes and about 30 transcripts were measured in routine experiments. However, it became clear that further reduction of the feature size would ultimately allow integration of entire genomes, which is the case today. In addition, array production and sample preparation methods underwent significant improvements resulting in robust and indispensable tools for routine applications in biomedical research [1].

In parallel microarray technologies for the genome wide analysis of single-nucleotide polymorphisms (SNPs) [2], copy number variation (CNV) [3] or DNA methylation [4] were successfully developed and marketed. Today a search for “DNA microarray” yields more than 50’000 PubMed entries which showcases the success of this technology and its wide range of applications. It is quite amazing that it took only 15 years of development time until multi-parallel interrogation of entire genomes became available for the research community. Currently, there are serious efforts ongoing to apply microarrays for protein based applications, such as epitope mapping [5]. However, this application is more challenging due to different properties of the ligand such as affinity or epitope folding.

Recently exceptionally efficient deep sequencing technologies became available at highly competitive prices. Measuring clone frequencies in bead libraries has the potential to replace or complement chip based fluorescence based transcript imaging in the future. One advantage of this approach is the possibility to detect any genomic transcript of an organism, provided the genome is available. Today many aspects of next-generation sequencing (NGS) remain to be solved. Although data generation can be fast, depending on the technology, the data analysis and processing have currently no user friendly solution, especially when multiple samples and conditions are part of the experiment. In addition, sample preparation is complex and certainly a source for artifacts which is reminiscent to the early days of microarray technology. Finally, the poor concordance of chip based transcript profiling experiments and NGS is inadequately understood in the research community [6]. In summary, I consider the use of microarrays is still the method of choice for routine experiments or studies that are carried out under GLP (good laboratory practice) regulations. The availability of user friendly commercial data analysis packages allows fast, robust and user-friendly data analysis and integration. Furthermore public expression databases such as GEO [7] allow comparative data analysis.

We welcome you to *Microarrays* and encourage submissions of interesting papers. We also welcome suggestions regarding the scope and content of the journal.
